# Two-level non-regular fractional factorial designs for public health studies

**DOI:** 10.1186/s12874-026-02784-6

**Published:** 2026-02-02

**Authors:** Ming-Chung Chang, Weng Kee Wong

**Affiliations:** 1 Institute of Statistical Science, Academia Sinica, Taipei, Taiwan; 2 Department of Biostatistics, University of California at Los Angeles, Los Angeles, CA 90095 USA

**Keywords:** Confounder, Factors’ interactions, Hidden projection property, Orthogonal array

## Abstract

This paper demonstrates the advantages of two-level non-regular fractional factorial designs for efficiently identifying influential factors in public health studies. We show how to construct smaller non-regular designs that can reliably estimate main effects and two-factor interactions in multi-factor studies, which are common in public health research. After some background information on non-regular designs and orthogonal arrays, we use real applications to support our claim. The applications include a behavioral intervention study (location/dates unreported), a drug combination trial (UCLA Micro Systems Laboratories, dates unreported), and a non-pharmaceutical Intervention study for influenza (agent-based simulation, dates unreported). We also provide resources on how to construct an appropriate non-regular design for a given problem. Our results from the three case studies show that smaller non-regular designs can identify key factors as reliably as the larger regular designs and they can require as much as 25% fewer runs, thereby reducing the study costs without sacrificing statistical precision in the inference The conclusion is that two-level regular designs are commonly used in public health studies but two-level non-regular designs can improve efficiency in screening multiple environmental, social, and behavioral factors, conserve resources and expedite discovery of evidence-based intervention effects. More specifically, two-level non-regular fractional factorial designs are more practical as they can be more flexible, cost efficient and provide just as reliable estimates as regular full or fractional factorial designs for a public health study with many interacting factors.

## Background

Full factorial experiments are widely used in industry and increasingly across disciplines to study the multifactorial effects of having several factors in an experiment. Many statistical books cover this topic, and an example is Montgomery [1]. There are also many tutorials or overview papers that provide a good description of factorial designs; an example is Pandis et al., [2] who discussed how to implement a factorial design for a dentistry study. The literature is replete with other applications, and some sample ones are using factorial designs for behavior therapy interventions [3], for testing abrasion by dentifrices in dental research [4], in the general design of clinical trials [5] and in education research, where a Bayesian paradigm was used to analyze the data from a factorial design [6]. 

Factorial designs can be used effectively to analyze the main effects and their interactions in a straightforward way. A main disadvantage of full factorial experiments is that the design becomes costly to implement when there are many factors and many runs are required. Fractional factorial experiments require the user to run only a subset of the factor level combinations from a full factorial design to make an inference; an example is Qamar et al., [7] where the goal was to extract cannabinoids from cannabis flowers by a carbon dioxide process using a fractional factorial design. Another recent and interesting application is Pashley and Bind [8], who employed a fractional factorial design to draw causal inference when there are multiple treatments in a study. Given an optimality criterion or a well-defined objective, one or more optimal fractional factorial designs can be determined [9]. Lundstedt et al. [10] provides an introduction to experimental design and optimization, along with screening methods for factorial and fractional factorial designs. More recently, Daraz et al. [11] and Zhang et al. [12] studied a specific optimality termed β-aberration for fractional factorial designs with number of factors higher than number of runs.

Orthogonal arrays (OAs) are also widely used in basic sciences, particularly in the engineering and manufacturing industry [13]. Lin and Stufken [14] reviews the fundamentals of OAs and discussed their applications in numerical integration, computer experiments, sub- sampling in big data, and error-correcting codes. In addition, the authors discussed recent developments, such as sliced OAs, nested OAs, strong OAs, and grouped OAs for a broader range of applications. Two recent applications of OA were used for designing a drug combination trial to treat tuberculosis [15], and for estimating a third-order response surface [16]. The latter was motivated after noting that in many industrial trials, the customary second-order model may not be adequate to fit the non-linearity of the underlying model, and third-order models should be considered and fitted using an orthogonal-array composite design, com- bined with a two-level OA and a four-level OA. There are many monographs that discuss these designs in detail and contain many real applications; for example, Wu and Hamada [17] is excellent as a resource.

We distinguish between regular fractional factorial designs and OAs. A regular fractional factorial design is generated from a full factorial by imposing algebraic defining relations (generators), which produce a highly structured aliasing pattern in which each effect is either completely clear or completely confounded with other effects. OAs provide a more general combinatorial framework: a regular fractional factorial design can be viewed as a special case of an OA, but many OAs do not correspond to regular designs. When such OAs are used as design matrices, they yield non-regular fractional factorial designs with more flexible aliasing patterns and, in many cases, additional run sizes that are not available under the regular fractional factorial designs.

Research in these designs is active and has been making tremendous advances in the last few decades to meet increasing design challenges for more complex studies. There is some use of factorial designs in clinical studies [3] but they are generally under-utilized. In particular, the newer and more flexible versions of fractional factorial designs are not used in public health research, despite their many benefits over current methods. For example, it is now known that serious diseases frequently require multiple drugs for treatment, and some can benefit from the right order of administration of the drugs [18, 19]. For instance, Yang et. al [20]. conducted an in vitro co-administration experiment with three drugs and found that the results suggest that the order of drugs can have an impact on treatment outcomes and applying all the drugs at the same time is not the most effective approach. These medical findings have inspired much current research in the design of order-of-addition experimental designs. Lin and Rios [21] provides the most updated review of research in order-of-addition experiments, and Stokes et al., [22] Rios and Lin [23], and Liu and Lan [24] are some recent papers in the area. In particular, the latter showed that a two-level component factorial design that combines a component orthogonal array design with a two-level fractional factorial design can more efficiently explore the order and dosage effect of the components and greatly reduce the number of experiments simultaneously.

Public health and epidemiology studies aim to improve population health by identifying the causes of diseases and developing effective strategies for prevention and treatment. These studies often involve complex systems in which multiple factors–such as biological, environ- mental, social, and behavioral—interact to affect health outcomes. Researchers often face practical challenges, including limited resources, ethical concerns, and diverse study populations. Well-designed studies are essential to address these challenges, support valid conclusions, and guide evidence-based decisions in both clinical and community settings [25–28]. 

To obtain meaningful information from a study with many factors, a key first step is to identify and remove those that have little or no effect on the outcome. Fractional factorial designs and orthogonal arrays are especially useful in this setting because they allow researchers to screen multiple factors, study their interactions, and efficiently focus on the ones that have the most impact. Phoa et al. [29] and Cavalcanti et al. [30] discussed the need of carefully considering and interpreting the interactions in the analysis of screening designs.

This paper proposes an even less discussed class of designs called non-regular fractional factorial designs for public health applications, with a focus on screening out inactive fac- tors. Non-regular designs, especially those generated from orthogonal arrays, have received growing attention because of their flexibility and useful statistical properties. One important feature of these designs is their hidden projection property [31]. In practical terms, even if the study later focuses on a smaller set of variables—for example, narrowing from many potential risk factors or treatment components to just a few—the design still allows accurate estimation of each main effect. It also preserves the ability to estimate how pairs of factors interact. This property is particularly valuable in medical, epidemiology, and public health research. Initial screening often involves many possible determinants of patient outcomes, but subsequent analyses concentrate on a smaller, clinically relevant subset without losing the ability to detect meaningful associations. In contrast, regular fractional factorial designs may suffer from complete aliasing among important effects when projected, due to their rigid defining relations. More details are available in Xu et al. [32] and the new and authoritative monograph on non-regular designs Cheng and Tang [33]. Further reading on non-regular fractional factorial designs and orthogonal arrays can be found in the design-of-experiments literature. For book-length treatments, we refer readers to Hedayat, Sloane, and Stufken [13], Mee [34], and the chapters on screening and non-regular designs in the Dean et al. [35] At the article level, important developments and catalogues of non-regular designs are given by Schoen [36], Schoen, Vo-Thanh, and Goos [37] and Schoen, Eendebak, and Nguyen [38]; together with the numerical recipes and software suggestions in this paper, these references provide practical starting points for constructing suitable non-regular designs in applications.

The goal of this work is to turn advances in non-regular orthogonal array designs into practical tools for public health and epidemiology, where resources are limited but many factors are of interest. Using two-level orthogonal arrays of strength two or three, we exploit their hidden projection properties so that, under the usual assumption that higher-order interactions are negligible, main effects and two-factor interactions remain estimable even when attention later narrows to subsets of factors. We provide simple rules and numerical recipes for selecting such non-regular designs with fewer runs than comparable regular fractional factorial designs, and we demonstrate through three case studies how they can replace commonly used regular designs in the health sciences.

This paper is organized as follows. The methods section briefly reviews non-regular fractional factorial designs and orthogonal arrays and then discusses the hidden projection properties of non-regular designs. The results section presents case studies illustrating their use and benefits, drawn from a web-based behavioral intervention trial on smoking cessation and a combinatorial drug therapy trial for Herpes Simplex Virus Type 1. The discussions section provides concluding remarks and directions for future research. Throughout, we use three real case studies from behavioral interventions, drug combination trials, and non-pharmaceutical interventions during an early pandemic influenza outbreak to demonstrate the value of non-regular fractional designs in addressing diverse public health challenges, offering advantages beyond those of conventional fractional factorial and orthogonal array designs.

### Intuitive overview of the design concepts

Before introducing the technical details, we briefly describe the main design concepts in intuitive terms.

In a two-level factorial design, each experimental condition corresponds to a combination of “high” or “low” levels of several factors. A regular fractional factorial design selects a subset of these combinations using simple algebraic rules. This structure is attractive for theory, but it limits the possible run sizes and can force investigators to use more experimental conditions than they would like. Non-regular designs relax these strict algebraic rules. They are often constructed from orthogonal arrays, which ensure that each level of a factor appears equally often and that pairs of factors occur in all level combinations. This flexibility allows for smaller run sizes and can reduce aliasing among important effects, at the cost of having a less tidy mathematical structure. In this paper we use the word “factor” in the design-of-experiments sense, to denote a column of the design matrix, and the word “treatment component” for the substantive elements of an intervention or study, such as counseling, medication, or a risk factor. Thus, risk factors are treated as one type of treatment component, and each treatment component corresponds to a factor in the experimental design.

The idea of “hidden projection” can also be explained informally. In practice, investigators may start with many potential factors but later focus on a smaller subset that turns out to be most relevant. A design with good hidden projection behaves well even after we ignore some columns of the design matrix. For public health and epidemiology studies, this means that investigators can begin with a relatively rich set of risk factors or intervention components, then narrow their attention to a smaller subset without losing the ability to obtain interpretable effect estimates from the original experiment.

To make the idea of hidden projection more concrete, consider a six-factor experiment with factors A–F. A common 16-run regular fractional factorial design with six two-level factors can be generated by taking four basic factors A, B, C, and D and defining E = AB and F = AC. In this regular design, the column for factor E is exactly the product of the columns for A and B. Consequently, if we later decide that only A, B, and E are of interest and fit a model with the three main effects and the two-factor interaction AB, the design matrix contains the columns for A, B, E, and AB, but the columns for E and AB are identical. The interaction AB is therefore completely aliased with the main effect of E, and these effects cannot be separated in the analysis.

Now suppose instead that we start from a two-level orthogonal array OA(12, 2^6^, 2) non-regular design for the same six factors. In this design, every pair of columns contains all four level combinations equally often, and the hidden projection property ensures that, under the usual assumption that higher-order interactions are negligible, the model matrix for any three factors has full rank for their main effects and two-factor interactions. In particular, if we again focus on factors A, B, and E, the columns for A, B, E, and AB are linearly independent, so all three main effects and the AB interaction remain estimable. This simple example illustrates how a non-regular design can retain estimability when interest narrows to a subset of factors.

**Fig. 1:**
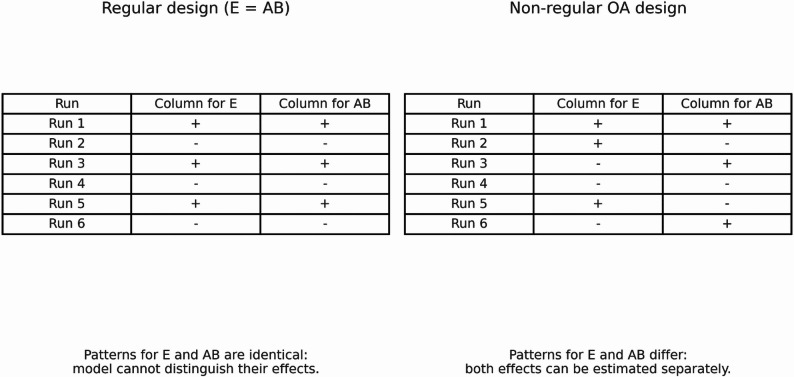
Simple worked example of aliasing versus hidden projection. Each column shows the pattern of “high” (+) and “low” (−) levels across six runs for the main effect of factor E and the interaction AB. Left: in a regular design with generator E = AB, the patterns in the two columns are identical, so the model cannot tell whether an observed effect is due to E or to the interaction AB. Right: in a non-regular OA design, the patterns for E and AB differ across runs, so their effects can in principle be estimated separately when the analysis focuses on factors A, B, and E

Figure 1 presents the same idea in a very concrete way. Each row represents one experimental condition and each “+” or “−” indicates whether the factor is at its high or low level in that run. In the regular design (left panel), the column for E and the column for the interaction AB have exactly the same pattern of “+” and “−”. As a result, any change in the outcome that follows this pattern could be attributed either to E or to AB, and the model cannot separate their effects. In the non-regular design (right panel), the patterns in the two columns are different, so the model can use the differences across runs to tease apart the main effect of E from the interaction AB. This is what we mean when we say that the non-regular design retains estimability after we focus on a subset of factors.

## Methods

Full factorial and fractional factorial designs are regular designs and are already widely used in screening studies in public health, epidemiology, and medical research to study multifactorial effects. We ask whether smaller and less costly studies can estimate main effects and selected two-factor interactions just as reliably. In this paper, we review the necessary preliminaries and illustrate the ideas using three examples: a behavioral intervention study, a drug combination trial at UCLA Micro Systems Laboratories, and a non-pharmaceutical influenza intervention study based on an agent-based (AB) simulation. We show in the Results section that two-level non-regular fractional factorial designs can efficiently identify influential factors and key two-factor interactions with similar precision to full or fractional designs. These non-regular designs are practical alternatives. They are flexible, time- and labor-efficient, and can reduce the required number of experimental conditions by up to 25% relative to larger regular designs. To promote their use in public health, epidemiology, and medical studies, we also provide numerical recipes and online tools for constructing user-specified two-level non-regular designs.

### Non-regular designs and orthogonal arrays

A full factorial design tests all possible combinations of factor levels but quickly becomes impractical as the number of factors increases. Regular fractional factorial designs reduce the number of runs by selecting a structured subset defined through specific defining re- lations, whereas non-regular designs select subsets without such constraints. High-quality non-regular designs often preserve the ability to estimate key effects when projected onto smaller subsets of factors, making them especially valuable for screening numerous variables under limited resources.

An *N* -run two-level orthogonal array of strength *t*, denoted OA(*N*, 2^*n*^, *t*), is an *N × n* matrix whose entries come from a two-level set (e.g., *{−*1, + 1*}*). Its defining property is that in every submatrix of *t* columns, all 2^*t*^ level combinations occur equally often. This combinatorial balance ensures that every effect involving up to *t* treatment components—such as single risk factors or interactions involving up to *t* factors—remains completely free of confounding. In early-stage clinical or public health screening studies, this means researchers can accurately assess the independent contribution of each factor to patient outcomes, even when testing many factors simultaneously. A library of orthogonal arrays is available at http://neilsloane.com/oadir/. All non-regular designs used in this study are orthogonal arrays of strength two, meaning that every possible combination of levels for any two factors occurs equally often. In clinical and public health research, this ensures that comparisons between any two treatment components—or risk factors—are made without bias from uneven representation, thereby improving the validity of conclusions drawn from early-phase trials or epidemiologic screening studies.

### Hidden projection properties of non-regular designs

One important advantage of non-regular designs is their superior hidden projection proper- ties, first introduced in Wang and Wu [31]. While regular designs have predictable and rigid aliasing structures based on their defining relations, non-regular designs—often constructed from orthogonal arrays—can show more favorable aliasing behavior when projected onto subsets of factors. The hidden projection property is particularly valuable in early-phase screening studies, where it is not yet clear which risk factors, biomarkers, or treatment components truly drive patient outcomes. This feature allows researchers to begin with a broad set of candidate variables and still obtain valid estimates for the most important ones once they emerge, without needing to redesign or repeat the study.

 Table 1 compares the hidden projection performance of an OA(12*,*2^6^*,*2) non-regular design with a 16-run regular design based on six two-level factors and defining relations *E*= *ABC*and *F*= *ACD*, leading to*I*= *ABCE*= *ACDF*= *BDEF*. The comparison focuses on the proportion of 3- and 4-factor projections that result in full-rank model matrices when considering only main effects and two-factor interactions. Here, “full rank” means that the design matrix contains enough independent information to estimate each main effect and two-factor interaction separately; no effect is a perfect linear combination of the others. Thus, 100% full-rank coverage for 4-factor projections implies that, regardless of which four factors later become the focus of analysis, all their main effects and pairwise interactions can in principle be estimated without complete aliasing. In contrast, 80% coverage indicates that for some choices of four factors, at least one two-factor interaction is aliased with other effects and cannot be disentangled, limiting what can be learned from the experiment.

**Table 1:** Projection performance of two 6-factor screening designs for a main-effects-plus-two-factor-interaction model. The table compares an OA(12, 2^6^, 2) non-regular design with a 16-run regular fractional factorial design with defining relations E = ABC and F = ACD. For each possible subset of 3 or 4 factors, we check whether the corresponding model matrix (including all main effects and two-factor interactions) is of full rank and report the percentage of subsets with full rank (“Full-rank (%)”). Higher percentages indicate better hidden projection properties

Subset Size	OA(12, 2^6, 2): Full-Rank (%)	16-Run Regular (6 factors): Full-Rank (%)
3 Factors	100%	100%
4 Factors	100%	80%

Remarkably, the OA(12, 2^6^, 2) design achieves 100% full-rank coverage for both 3-factor and 4-factor subsets. In contrast, although the regular design also achieves 100% coverage for 3-factor projections, it performs worse for 4-factor projections. Among the six choose 4 possibilities, that result in the 15 possible 4-factor subsets from the regular design, only 12 yield a full-rank model matrix under the considered model. The remaining 3 subsets—*{A*,* B*,* C*,* E}*, *{A*,* C*,* D*,* F}*, and *{B*,* D*,* E*,* F}*—fail to achieve full rank due to aliasing introduced by the defining relations. These results demonstrate that the non- regular design not only matches the regular design in lower-dimensional projections but also outperforms it in higher-dimensional ones—even though it requires only 12 runs, compared to 16 runs for the regular design.

This result highlights the hidden strength of OA-based non-regular designs. In practical terms, this means that experimenters using non-regular designs are more likely to retain the ability to identify important factors and interactions when adapting or narrowing their studies. This is especially valuable in public health, epidemiology and medical research, where study goals and conditions often change during the study period. In addition, the smaller number of runs provides logistical and ethical advantages in settings where resources are limited or where experimentation must be done with care.

In fact, the result above is not accidental. According to Corollary 15.9 in Cheng [39], for an OA(*N*, 2^*n*^, 2), if *N* is not a multiple of 8 and *n ≥* 4, then the projection of such an array onto any four factors ensures that all main effects and two-factor interactions are estimable, assuming that higher-order interactions are negligible. This result shows that even though strength-2 orthogonal arrays are not designed to fully balance three-factor interactions, they still have enough structure to support meaningful inference in moderately sized subsets. This hidden projection property is especially useful in practical applications, such as screening studies, where it is not known in advance which factors will be important in later stages. Consequently, strength-2 orthogonal arrays are practical and efficient choices for initial screening designs.

In addition to orthogonal arrays with strength 2, orthogonal arrays with strength 3, denoted as OA(*N*, 2^*n*^, 3), are known to have an even stronger form of the hidden projection property. According to Corollary 15.12 in Cheng [39], if *n ≥* 5 and *N* is not a multiple of 16, then the projection onto any five columns ensures that all main effects and two- factor interactions are estimable, assuming higher-order interactions are negligible. Table 2 summarizes sufficient conditions under which all main effects and two-factor interactions are estimable in projections, assuming higher-order interactions are negligible.

**Table 2:** Hidden projection properties of two-level orthogonal arrays OA(N, 2^n^, t). For each strength t = 2 or 3, the table lists simple sufficient conditions on the number of runs N under which projections onto subsets of a given size yield model matrices in which all main effects and two-factor interactions are estimable without aliasing, assuming higher-order interactions are negligible. “Projection subset size” denotes the number of factors retained in the subset

Strength	Condition on *N*	Projection Subset Size	Estimable Effects
2	*N* not a multiple of 8	Any 4 factors	Main effects and 2FIs
3	*N* not a multiple of 16	Any 5 factors	Main effects and 2FIs

## Results

Public health and epidemiology studies often aim to identify effective components of interventions that target behavioral, clinical, or environmental factors. These studies often involve multiple factors, including messaging types, delivery methods, dosage, timing, and treatment combinations. In this section, we present three real examples from public health studies and show how non-regular fractional factorial designs can improve the implemented design in each application in different ways.

### Application 1: project quit and an OA(12, 2^6^, 2) design

The Project Quit study described in Nair et al. [27] was a web-based behavioral intervention targeting smoking cessation. Although the trial location and dates were not reported, it enrolled 1848 participants to identify key components influencing quitting behavior. Six two-level intervention components (factors) were evaluated. Factor *A* was *type of exposure*, comparing a single, large set of materials with multiple correspondences over several weeks. Factor *B* was *outcome expectation depth*, where the high-depth group received individual- ized feedback and advice, while the low-depth group received general motivational content. Factor *C* was *success story depth*, with the high-depth group reading stories tailored to their sociodemographic background, and the low-depth group receiving gender-tailored stories. Factor *D* was *efficacy expectation*, contrasting specific feedback on major barriers to quitting (high-depth) with broader, general advice (low-depth). Factor *E* was *source person- alization level*, where high-depth participants saw personalized materials including photos and messages from a health team, while low-depth participants saw only a photo. Fac- tor *F* was *message framing*, comparing gain-framed messages (highlighting the benefits of quitting) to loss-framed messages (emphasizing the harms of continued smoking). Due to practical constraints, a regular fractional factorial design with 16 experimental conditions was used in Nair et al., [27] with defining relations *E* = *ABC* and *F* = *ACD*. This structure allowed the researchers to estimate main effects and selected two-factor interactions under the assumption that higher-order interactions were negligible.

Based on the discussion in the methods section, an appealing alternative is the use of a non-regular design based on an orthogonal array OA(12, 2^6^, 2), given in Table 3.

**Table 3:** Candidate OA(12, 2^6^, 2) non-regular design for the Project Quit behavioral intervention study (Application 1). Rows correspond to the 12 experimental conditions and columns A–F correspond to the six two-level intervention components described in the text. Entries are coded as − 1 and + 1 for the low and high levels, respectively. Because this orthogonal array has strength two, every pair of columns contains all four level combinations equally often, ensuring that all main effects and two-factor interactions are unconfounded in any two-factor projection

A	B	C	D	E	F
1	1	1	1	1	*−*1
*−*1	1	1	1	*−*1	1
*−*1	*−*1	1	1	1	*−*1
1	*−*1	*−*1	1	1	1
*−*1	1	*−*1	*−*1	1	1
*−*1	*−*1	1	*−*1	*−*1	1
*−*1	*−*1	*−*1	1	*−*1	*−*1
1	*−*1	*−*1	*−*1	1	*−*1
*−*1	1	*−*1	*−*1	*−*1	1
1	*−*1	1	*−*1	*−*1	*−*1
1	1	*−*1	1	*−*1	*−*1
*−*1	*−*1	*−*1	*−*1	*−*1	*−*1

This design includes only 12 runs—25% fewer than the regular design used in Project Quit—yet retains strength 2. Additionally, under the same assumption that higher- order interactions are negligible, the hidden projection property of the OA-based non-regular design ensures unbiased estimation of main effects and two-factor interactions when projected onto any four factors.

We note that when projected onto four factors, the 16-run regular design may result in fully confounded pairs of two-factor interactions due to the two defining relations: *E* = *ABC* and *F* = *ACD*. Even with a 64-run full factorial design, which permits estimation of all factorial effects, many effects are likely insignificant at the screening stage, leading to unnecessary waste. Thus, replacing the original 16-run regular design with the OA(12, 2^6^, 2) non-regular design would reduce the number of experimental conditions from 16 to 12 while preserving estimability of all main effects and the selected two-factor interactions. Investigators could keep the per-condition sample size unchanged, leading to a 25% reduction in total sample size and implementation cost, or keep the total sample size fixed and allocate more participants to each of the 12 conditions to gain precision. Our focus in this application is on this reduction in the number of distinct treatment combinations and the associated gains in design efficiency.

### Application 2: project HSV-1 and an OA(28, 2^6^, 2) design

Jaynes et al. [40] presents a systematic approach to evaluating combinatorial drug therapies for Herpes Simplex Virus Type 1 (HSV-1), a widespread pathogen associated with oral lesions, encephalitis, and increased susceptibility to HIV infection. The experiments were conducted at the UCLA Micro Systems Laboratories (specific dates unknown). Motivated by the lim itations of monotherapy—such as drug resistance and toxicity observed with agents like Acyclovir—the authors employed fractional factorial designs to efficiently assess six antiviral candidates: Interferon-alpha, Interferon-beta, Interferon-gamma, Ribavirin, Acyclovir, and TNF-alpha. A resolution VI 2^6*−*1^ fractional factorial design with center points was used to screen for main effects and interactions.

In contrast to regular fractional factorial designs of resolution VI, which assume higher- order interactions (e.g., three-factor interactions) are negligible, we propose using an OA with four fewer runs than the 2^6*−*1^ design: OA(28, 2^6^, 2), as shown in Table 4. This OA was generated using the oa.design function from the DoE.base package in R [41]. The OA ensures pairwise unconfounded among all main effects and reduces confounding among two-factor interactions.

**Table 4:** Candidate OA(28, 2^6^, 2) non-regular design for the HSV-1 drug combination trial (Application 2). Rows correspond to the 28 experimental runs and columns A–F represent the six antiviral agents (interferon-alpha, interferon-beta, interferon-gamma, ribavirin, acyclovir, and TNF-alpha), coded at two levels (− 1 = low, + 1 = high). As a strength-two orthogonal array, every pair of columns contains all four level combinations equally often, providing pairwise unconfounded main effects and reduced aliasing among two-factor interactions compared with the original regular fractional factorial design

Run	A	B	C	D	E	F
1	*−*1	1	*−*1	1	*−*1	*−*1
2	*−*1	*−*1	1	1	*−*1	1
3	1	*−*1	*−*1	1	1	*−*1
4	*−*1	*−*1	1	*−*1	*−*1	1
5	1	*−*1	1	1	*−*1	*−*1
6	1	*−*1	1	1	1	1
7	1	1	*−*1	*−*1	*−*1	1
8	*−*1	1	*−*1	1	*−*1	1
9	*−*1	1	*−*1	1	1	*−*1
10	1	*−*1	*−*1	*−*1	1	1
11	*−*1	*−*1	*−*1	*−*1	*−*1	*−*1
12	1	1	1	1	*−*1	1
13	1	1	1	1	1	*−*1
14	1	1	1	*−*1	1	1
15	*−*1	1	1	*−*1	*−*1	1
16	*−*1	1	*−*1	*−*1	1	*−*1
17	1	*−*1	*−*1	*−*1	*−*1	1
18	1	1	*−*1	1	1	1
19	*−*1	1	1	1	1	1
20	1	*−*1	*−*1	1	*−*1	*−*1
21	*−*1	*−*1	1	*−*1	1	*−*1
22	1	*−*1	1	*−*1	1	*−*1
23	*−*1	*−*1	1	1	*−*1	*−*1
24	*−*1	*−*1	*−*1	1	1	1
25	*−*1	*−*1	*−*1	*−*1	1	1
26	1	1	*−*1	*−*1	*−*1	*−*1
27	*−*1	1	1	*−*1	1	*−*1
28	1	1	1	*−*1	*−*1	*−*1

### Application 3: project NPI and a 420-run paley design

Martinez and Das [42] addressed the urgent need for effective non-pharmaceutical interventions (NPIs) during the early stages of a pandemic influenza outbreak, when vaccines and antiviral treatments are not yet widely available. Their study emphasized the importance of NPIs as essential tools for mitigating disease transmission, and they include case isolation, household quarantine, school closures, and workplace shutdowns. To manage the complexity arising from the high dimensionality of intervention parameters and their potential interactions, the authors adopted a simulation-based experimental design framework. Specifically, they implemented a 2^16*−*7^ fractional factorial design to systematically evaluate 16 key NPI-related factors—each at two levels—reducing the total number of required simulation runs from 65,536 to 512. These factors included global infection thresholds, delays in policy implementation, compliance rates across different population segments, and the duration and triggering criteria for school and workplace closures.

As noted in Martinez and Das [42], the 2^16*−*7^ design ensures entire unconfoundedness between main effects and two-factor interactions, but not among the two-factor interactions themselves. As a result, when the design is projected onto a subset of four factors, it is generally not possible to estimate all two-factor interactions simultaneously. The methods section discusses an alternative approach based on non-regular designs constructed from orthogonal arrays. In particular, we consider a class of non-regular designs known as *Paley designs*, introduced by Paley [43]. Theorem 15.13 in Cheng [39] establishes that any Paley design of size at least 12possesses the hidden projection property. Paley designs can be readily generated using the paley function in the survey package in R [41]. We use the paley function to generate a Paley design with 420 runs for this application. Compared to the 2^16*−*7^ design, the Paley design achieves approximately an 18% reduction in the number of runs while offering superior projection properties.

In addition to the Paley design, *quaternary code designs* provide another rich source of highly efficient non-regular two-level designs. Very loosely speaking, these designs start from special “quaternary codes” defined on four symbols (0, 1, 2, 3) and then use a Gray map to convert them into two-level designs that can accommodate many factors with very high generalized resolution; see Xu and Wong (2007, Chap. 4) for a detailed construction and extensive catalogs. Subsequent work by Phoa and Xu (2009), Zhang, Phoa, Mukerjee and Xu (2011), and Mukerjee and Tang (2013) further develops quarter-fraction quaternary designs and complementary design theory, making quaternary code designs a useful option when very high-resolution designs with 128 or 256 runs are desired.

## Conclusions

In public health and epidemiology studies, experiments often involve many factors but are constrained by limited resources. Non-regular fractional factorial designs based on orthogonal arrays can be more efficient than regular designs because of their hidden projection property, which preserves the ability to estimate main effects and two-factor interactions even when interest later narrows to a subset of factors. In clinical trials or public health intervention studies, this means that if only certain treatment components or risk factors turn out to be important, researchers can still obtain unbiased estimates for these key effects without having planned for them in advance. This feature is particularly valuable in early-phase screening studies, where higher-order interactions are usually negligible and the primary goal is to identify promising treatment components or risk factors using fewer experimental runs.

This study is for two-level designs and assumes homogeneous individuals. Through three case studies, we demonstrated that non-regular designs improved or replaced regular fractional factorial designs. An OA(12, 2^6^, 2) matched the estimability of main effects and two-factor interactions while using 25% fewer runs; an OA(28, 2^6^, 2) required fewer runs than a resolution VI 2^6−1^ design and reduced aliasing among two-factor interactions; and a 420-run Paley design achieved better hidden projection with about 18% fewer runs than a 2^16−7^ design. Overall, these results show that non-regular designs can screen many factors more efficiently while reducing burden and preserving clear interpretation of key effects.

Future work can proceed in two directions. First, asymmetric orthogonal arrays with two and three levels would allow quadratic effects for quantitative factors to be studied, helping to detect factors with weak linear but strong nonlinear influences. Second, blocked non-regular designs are needed to handle heterogeneity in age, sex, socioeconomic status, or baseline health by grouping similar individuals, controlling nuisance variation, and preserving favorable projection and aliasing properties for greater precision and practical applicability. For methods to generate mixed-level non-regular designs accommodating diverse heterogeneity structures, see Chang [44]. 

## Data Availability

No new data was collected and all data used in the paper are publicly available.
